# Altered glucose metabolism and hypoxic response in alloxan-induced diabetic atherosclerosis in rabbits

**DOI:** 10.1371/journal.pone.0175976

**Published:** 2017-04-14

**Authors:** Yunosuke Matsuura, Atsushi Yamashita, Yan Zhao, Takashi Iwakiri, Kazuaki Yamasaki, Chihiro Sugita, Chihiro Koshimoto, Kazuo Kitamura, Keiichi Kawai, Nagara Tamaki, Songji Zhao, Yuji Kuge, Yujiro Asada

**Affiliations:** 1 Department of Pathology, Faculty of Medicine, University of Miyazaki, Miyazaki, Japan; 2 Department of Internal Medicine, Faculty of Medicine, University of Miyazaki, Miyazaki, Japan; 3 Department of Tracer Kinetics and Bioanalysis, Graduate School of Medicine, Hokkaido University, Sapporo, Japan; 4 Department of Biochemistry and Microbiology, Faculty of Pharmaceutical Sciences, Kyusyu University of Health and Welfare, Nobeoka, Japan; 5 Frontier Science Research Center, University of Miyazaki, Miyazaki, Japan; 6 Faculty of Health Sciences, Institute of Medical, Pharmaceutical and Health Sciences, Kanazawa University, Kanazawa, Japan; 7 Biomedical Imaging Research Center, University of Fukui, Fukui, Japan; 8 Department of Nuclear Medicine, Graduate School of Medicine, Hokkaido University, Sapporo, Japan; 9 Department of Integrated Molecular Imaging, Graduate School of Medicine, Hokkaido University, Sapporo, Japan; 10 Central Institute of Isotope Science, Hokkaido University, Sapporo, Japan; National Cerebral and Cardiovascular Center, JAPAN

## Abstract

Diabetes mellitus accelerates atherosclerosis that causes most cardiovascular events. Several metabolic pathways are considered to contribute to the development of atherosclerosis, but comprehensive metabolic alterations to atherosclerotic arterial cells remain unknown. The present study investigated metabolic changes and their relationship to vascular histopathological changes in the atherosclerotic arteries of rabbits with alloxan-induced diabetes. Diabetic atherosclerosis was induced in rabbit ilio-femoral arteries by injecting alloxan (100 mg/kg), injuring the arteries using a balloon, and feeding with a 0.5% cholesterol diet. We histologically assessed the atherosclerotic lesion development, cellular content, pimonidazole positive-hypoxic area, the nuclear localization of hypoxia-inducible factor-1α, and apoptosis. We evaluated comprehensive arterial metabolism by performing metabolomic analyses using capillary electrophoresis-time of flight mass spectrometry. We evaluated glucose uptake and its relationship to vascular hypoxia using ^18^F-fluorodeoxyglucose and pimonidazole. Plaque burden, macrophage content, and hypoxic areas were more prevalent in arteries with diabetic, than non-diabetic atherosclerosis. Metabolomic analyses highlighted 12 metabolites that were significantly altered between diabetic and non-diabetic atherosclerosis. A half of them were associated with glycolysis metabolites, and their levels were decreased in diabetic atherosclerosis. The uptake of glucose evaluated as ^18^F-fluorodeoxyglucose in atherosclerotic lesions increased according to increased macrophage content or hypoxic areas in non-diabetic, but not diabetic rabbits. Despite profound hypoxic areas, the nuclear localization of hypoxia-inducible factor-1α decreased and the number of apoptotic cells increased in diabetic atherosclerotic lesions. Altered glycolysis metabolism and an impaired response to hypoxia in atherosclerotic lesions under conditions of insulin-dependent diabetes might be involved in the development of diabetic atherosclerosis.

## Introduction

Atherosclerosis is the cause of most cardiovascular diseases (CVD), and diabetes mellitus (DM) accelerates progression of atherosclerotic disease and the subsequent occurrence of atherothrombosis [1–4]. In general, the quantity and quality of atherosclerotic plaques are major determinants of the incidence of cardiovascular events (CVE). A recent series of imaging and pathological studies has confirmed that plaque burden is increased in patients with DM, compared with those without DM [5,6]. On the other hand, whether or not diabetic and non-diabetic atherosclerotic lesions histologically differ is unclear [7]. These lines of evidence indicate that the underlying mechanisms of plaque progression and the subsequent onset of CVE in patients with DM remains unknown. Medical intervention and efforts to lower glucose levels have failed to prevent the development and progression of diabetes associated with macroangiopathy in the clinical setting [8,9]. Therefore, innovative approaches are urgently required to prevent atherosclerosis in patients with DM.

Persistent hyperglycemia seems to be responsible for the development of atherosclerotic lesions. Increased glucose flux through various metabolic pathways is thought to generate toxic precursors that contribute to vascular cell damage. Harmful metabolic pathways include the formation of advanced glycation end products, protein kinase C activation and disrupted polyol and hexosamine pathways [10]. Dyslipidemia due to increased lipolysis might also contribute to the progression of atherosclerosis. Therefore, DM is considered to largely affect vascular cell metabolism, which leads to the development of atherosclerosis. Identifying changes in metabolic pathways and metabolites in atherosclerotic lesions under the conditions imposed by DM would be critical to understanding the underlying mechanisms of atherogenesis in patients with DM and might provide the means to develop novel therapeutic targets.

Metabolomics can simultaneously identify levels of endogenous metabolites in biological samples, such as blood, urine and tissues, and analytical datasets provide comprehensive information in a snapshot of metabolic status during dynamic disease processes. Recent studies using metabolomics profiling have revealed serum levels of branched-chain and aromatic amino acids, including leucine, isoleucine, valine, phenylalanine, and tyrosine, are closely associated with the risk of future diabetes [11, 12]. However, metabolic alterations in diabetic atherosclerosis have not been reported.

We assessed metabolomics and the effects of hypoxia on atherosclerotic lesions in rabbits with alloxan-induced diabetes to identify metabolic changes in diabetic atherosclerosis and its relationship to vascular pathology.

## Materials and methods

### Diabetic rabbit atherosclerotic model

The animal research protocol of the present study conformed to the Guide for the Care and Use of Laboratory Animals published by the US National Institutes of Health, and was approved by the Animal Care Committee of Miyazaki University (2010–541). Experiments proceeded under aseptic conditions and general anaesthesia induced via subcutaneous administration of medetomidine (0.16mg/kg) and butorphanol tartrate (0.4mg/kg) and intravenous infusion of midazolam (0.6 mg/kg). Diabetic rabbits were generated from Japanese white rabbits initially weighing 2.5–3.5 kg using a single dose of alloxan [13]. Atherosclerotic lesions were produced by feeding the rabbits with a high-cholesterol diet and denuding the endothelium of the femoral arteries in diabetic and non-diabetic rabbits. In detail, a single 100-mg/kg dose of alloxan monohydrate (Sigma, St Louis, MO, USA) dissolved in 10 mL of sterile saline was injected to induce diabetes. Non-diabetic rabbits were injected with saline. Initial hypoglycemia after alloxan injection was prevented by an immediate injection of 10 mL of 20% glucose and the provision of 5% glucose in the drinking water ad libitum for 24 hours. Thereafter, blood glucose levels were monitored weekly and rabbits with fasting plasma glucose levels > 200 mg/dL during the entire experimental period were defined as diabetic and included in this study. One week after injection with alloxan or saline, the rabbits were started on the 0.5% cholesterol diet for four weeks. One week after starting the cholesterol diet, the endothelium of the ilio-femoral arteries was denuded using an angioplasty balloon catheter as described [14]. At baseline and at five weeks after alloxan or saline injection, the rabbits were weighed and levels of insulin, total cholesterol, triglyceride and free fatty acid were measured after an overnight fast.

### Metabolomic profiling of vascular tissues using Capillary Electrophoresis Time-Of-Flight Mass Spectrometry (CE-TOFMS)

#### Tissue preparation

The rabbits were sacrificed with intravenous infusion of pentobarbital (60 mg/kg). Soft tissues surrounding isolated rabbit arteries were removed and the arteries were immediately frozen in liquid nitrogen and stored at -80°C. Arteries were weighed and then the tissues (30mg) were added to 500 μL of methanol containing 50 μM of internal standard and homogenized using a tissue disrupter. Homogenates were mixed with 200 μL of Milli-Q water and 500 μL of chloroform and then separated by centrifugation at 2300 × g for 5 min at 4°C. The upper aqueous layer (400 μL) was passed through a filter (5-kDa cutoff; Millipore) by centrifugation at 9,100 g for 120 min at 4°C to remove proteins. The filtrates were lyophilized and suspended in 50 μL of Milli-Q water for metabolome analysis.

#### Instrumentation

Metabolomes were profiled by CE-TOFMS using an Agilent CE Capillary Electrophoresis System equipped with a 6210 Time-of-Flight mass spectrometer, an 1100 isocratic HPLC pump, a G1603A CE-MS adapter kit and a G1607A CE-ESI-MS sprayer kit (all from Agilent Technologies, Waldbronn, Germany), and this system was controlled by G2201AA ChemStation software version B.03.01 for CE (Agilent Technologies).

#### CE-TOFMS conditions

Cationic metabolites were analyzed using a fused silica capillary (50 μm i.d. × 80 cm length), with H3301-1001 cation electrophoresis buffer (Human Metabolome Technologies, Tsuruoka, Yamagata, Japan) as the electrolyte. Samples were injected at a pressure of 50 mbar for 10 sec. The applied voltage was set at 27 kV. Electrospray ionization-mass spectrometry proceeded in positive-ion mode, and the capillary voltage was set at 4,000 V. Samples were scanned by the spectrometer between 50 and 1,000 m/z [15].

Anionic metabolites were analyzed using a fused silica capillary (50 μm i.d. × 80 cm length), with H3302-1021 anion electrophoresis buffer (Human Metabolome Technologies) as the electrolyte. Samples were injected at a pressure of 50 mbar for 25 sec. The applied voltage was set at 30 kV. Electrospray ionization-mass spectrometry proceeded in negative-ion mode, and the capillary voltage was set at 3,500 V. Samples were scanned by the spectrometer between 50 and 1,000 m/z [15].

#### Processing and analysis of metabolites data

Raw data derived by CE-TOFMS were automatically processed by the proprietary software MasterHands [11]. Signal peaks corresponding to isotopomers, adduct ions and other product ions of known metabolites were excluded. All signal peaks potentially corresponding to authentic compounds were extracted and their migration times (MT) were normalized to internal standards. Thereafter, peaks were aligned in accordance with m/z values and normalized MT. Finally, peak areas were normalized against those of the respective internal standards for cations, anions, methionine sulfone and D-camphol-10-sulfonic acid. Values for relative areas were further normalized by sample weight. Annotation tables were produced from values of standard compounds and aligned with datasets according to similar m/z values and normalized MT.

### Hypoxia and ^18^F-fluorodeoxyglucose (FDG) uptake

We investigated ^18^F-FDG uptake as described with minor modifications [15]. Diabetic and non-diabetic rabbits (n = 6 in each) were fasted for four hours and then infused with ^18^F-FDG (average, 207 MBq/rabbit) and 60 mg/kg of the hypoxia marker, pimonidazole (Hypoxyprobe-1; Hypoxyprobe, Inc., Burlington, MA, USA). Two hours later, amounts of radioactivity in the femoral arteries, and blood were measured using a 1480 WIZARD 3 well-type γ-counter (Wallac Co. Ltd., Turku, Finland). The results were calculated as (%ID/g) × kg.

Atherosclerotic and control ilio-femoral arteries were excised, cut into 8 to 12 segments, embedded in Tissue-Tek (Sakura, Tokyo, Japan) and frozen. Ten and 5-μm consecutive slices were prepared for autoradiography and histological analysis, respectively, and then 10-μm cryostat sections were exposed on phosphor imaging plates (Fuji Imaging Plate BAS-SR 2025, Fuji Photo Film Co. Ltd., Tokyo, Japan) for 12 h together with a set of calibrated standards. The plates were then scanned using a Fuji Bio-imaging Analyzer BAS-5000 (Fuji Photo Film) with an internal resolution of 25-μm and the images were assessed using Multi Gauge Ver. 3.0 image analysis software (Fuji Photo Film). The amount of radioactivity in each image is expressed as photo-stimulated luminescence (PSL) per unit area (PSL = a × D × t, where a is a constant, D is the amount of radioactivity exposed on the imaging plate, and t is exposure duration). All PSL values/mm^2^ from the arterial tissue were converted to ratios (%) of the activity of the standard injected dose/mm^2^ of lesion area (% ID/m^2^). Data were normalized according to the weight of each rabbit (% ID/m^2^ × kg).

### Histopathology and immunohistochemistry

Serial frozen sections (5-μm thick) were stained with hematoxylin and eosin (HE) and Victoria Blue (VB). Sections were immunohistochemically assessed using mouse monoclonal anti-α-smooth-muscle-cell actin for smooth muscle cells (SMC) (Dako, Glostrup, Denmark), RAM-11 mouse monoclonal anti-rabbit macrophages (Dako), mouse monoclonal anti-pimonidazole (Hypoxyprobe), and mouse monoclonal hypoxia inducible factor (HIF)-1α (Abcam, Cambridge, UK) as primary antibodies. Horseradish peroxidase activity was visualized by staining with 3, 3’-diaminobenzidine tetrahydrochloride and counterstaining with Meyer’s hematoxylin. The negative control was stained with universal negative control mouse immunoglobulins (Dako) instead of primary antibodies.

### Morphometric analysis

Vessel areas and positive immunostained parts of whole cross-sectional areas of vascular tissues were quantified using a color-imaging morphometry system (Win Roof, Mitani, Fukui, Japan). Immunopositive areas are expressed as ratios (%) of vessel areas. Two investigators (T. I. and C. S.) accomplished all quantitative analyses in a blinded manner.

### Western blotting

Nuclear proteins of rabbit diabetic and non-diabetic atherosclerotic arteries were extracted using a mammalian nuclear protein extraction reagent (Thermo Fisher Scientific Inc., Waltham, MA, USA) with 1% protease inhibitor cocktail (Thermo Fisher). Protein concentrations were determined using DC protein assay kits (Biorad, Hercules, CA, USA).

The nuclear translocation of HIF-1α in diabetic and non-diabetic atherosclerosis was determined by sodium dodecyl sulfate-polyacrylamide gel electrophoresis (SDS-PAGE) using 10% gels (Technical Frontier Co., Tokyo, Japan). Samples were incubated with sample buffer containing SDS and 2-mercaptoethanol for three minutes at 98°C before SDS-PAGE. Separated proteins were electrophoretically transferred to Immobilon membranes (Millipore, Billerica, MA, USA). Non-specific binding was blocked overnight with 5% skimmed milk, and then the separated proteins were incubated with anti-HIF-1α antibody (Abcam) followed by horseradish peroxidase conjugated anti-mouse IgG (Nacalai Tesque Inc., Kyoto, Japan). After stripping the antibodies, the membrane was reprobed with anti-lamin A/C (Sigma) and the secondary antibody. The proteins of interest were detected by chemiluminescence using a LAS-4000 Lumino image analyzer (Fuji).

### TUNEL assay

The numbers of apoptotic cells in the ilio-femoral arteries of diabetic and non-diabetic rabbits were determined using terminal deoxynucleotidyl transferase-mediated deoxyuridine triphosphate nick end labeling (TUNEL) assays. Apoptotic cells in arterial samples that had been fixed in 4% paraformaldehyde and embedded in paraffin were visualized using *in situ* apoptosis detection kits (Takara Bio Inc., Kusatsu, Japan) and data are expressed as numbers of positive nuclei per 10,000 μm^2^. Double immunofluorescence emission by apoptotic cells and hypoxia was determined using TUNEL assays, fluorescein isothiocyanate-labeled anti-BrdU antibody (Apo-BrdU *in situ* DNA fragmentation assay kits [BioVison Inc., Milpitas, CA, USA]), and Dylight^™^549 fluorophore-labeled anti-pimonidazole antibody (Hypoxyprobe).

### Statistical analysis

Data were analyzed using SampleStat (Human Metabolome Technologies Inc., Tsuruoka, Japan) for Principal Component Analysis (PCA), PeakStat (Human Metabolome Technologies) for Hierarchical Cluster Analysis (HCA), JMP 7.0 (SAS Institute Inc., Cary, NC, USA) and Graph Pad Prism 5.01 (GraphPad Software Inc., San Diego, CA). Values are expressed as means with standard deviation (SD). Variables between or among groups were compared using Mann-Whitney U test or One-way ANOVA with Bonferroni’s multiple comparisons test. Relationships between factors were evaluated using Spearman’s test. P values < 0.05 were considered statistically significant.

## Results

### Body weight and blood parameters in diabetic and non-diabetic rabbits fed with diet containing 0.5% cholesterol

Diabetic rabbits were generated using a single 100-mg/kg dose of alloxan monohydrate. S1 Table shows body weight (BW) and serum levels of glucose, insulin, total cholesterol and triglyceride in blood samples obtained after an overnight fast at baseline and after five weeks from non-diabetic (n = 10) and diabetic (n = 10) rabbits fed with the cholesterol diet. None of the parameters differed between the two groups at baseline. At five weeks later, however, BW and insulin levels were significantly lower, and mean values of glucose, total cholesterol and triglyceride were ~ 3.5-, 2.0- or 8.0-fold higher, respectively, after five weeks in diabetic, than non-diabetic rabbits.

### Vascular metabolism profiling using CE-TOFMS

Metabolomic analysis proceeded using CE-TOFMS in; non-diabetic, non-atherosclerotic arteries (n = 5; control), non-diabetic and diabetic atherosclerotic arteries (n = 5 each). Atherosclerotic lesions were produced by feeding the rabbits with a high-cholesterol diet and denuding the endothelium of the ileo-femoral arteries in diabetic and non-diabetic rabbits. Overall, 211 peaks (122 cations and 89 anions) were identified based on m/z values and migration times, in the arterial tissues. In detail, 155, 184 and 205 peaks were detected in control, and in non-diabetic and diabetic atherosclerotic samples, respectively. We also absolutely quantified 110 metabolites including glycolytic and citric acid cycle intermediates, amino acids and purine nucleoside phosphates.

**Fig 1:**
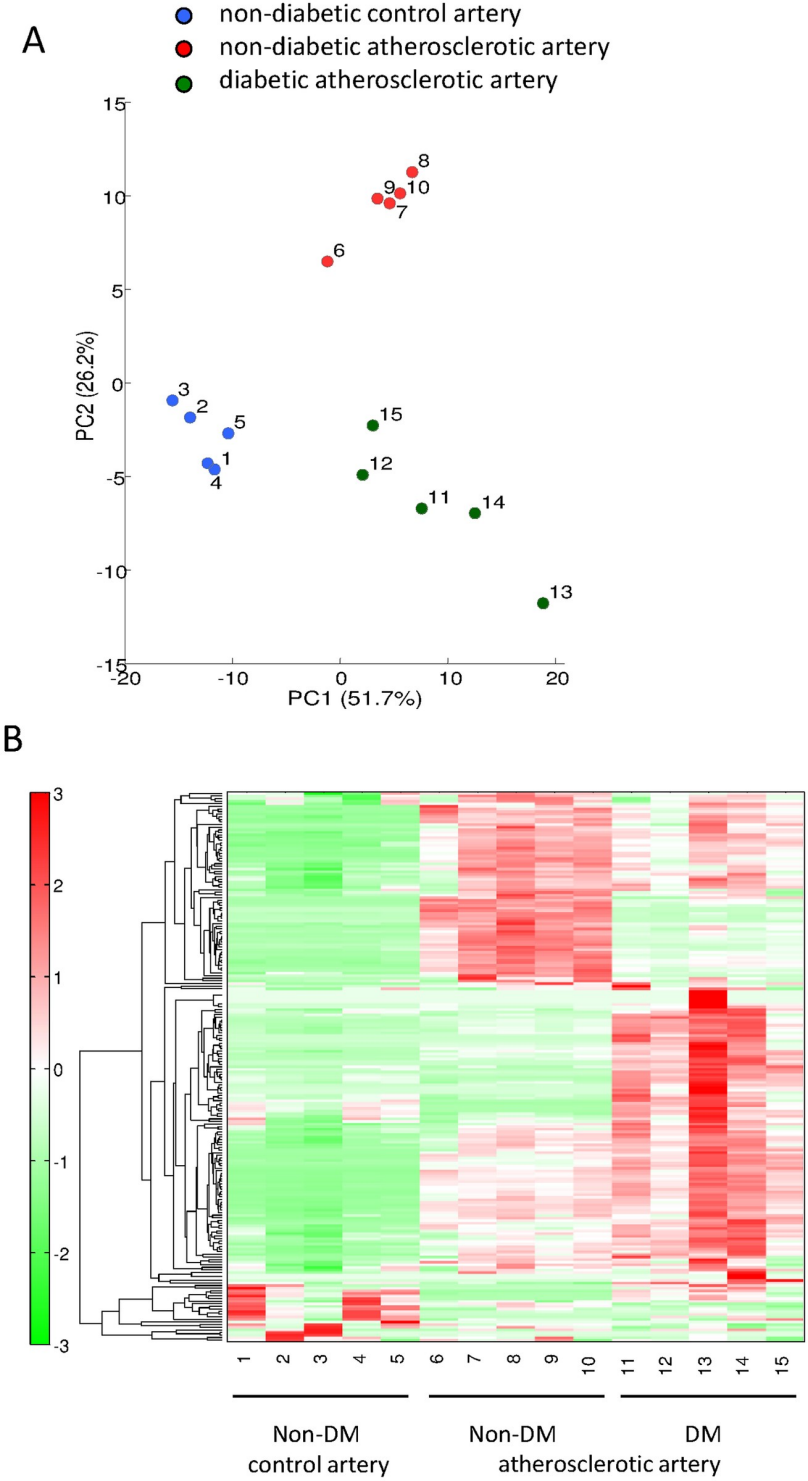
Metabolomic analysis of arterial metabolites in rabbits with and without alloxan-induced diabetes. A. Principal Component Analysis (PCA) discriminates metabolic features among non-diabetic, control arteries (1–5, blue), non-diabetic atherosclerotic arteries (6–10, red), and diabetic atherosclerotic arteries (11–15, green). S2 Table shows factor-loading values for PC1 and PC2 on score plots. B. Representative heat map assessed using hierarchical clustering analysis shows metabolic differences among groups. S3 Table shows original data for each metabolite.

Fig 1A shows the results of principal component analysis (PCA). The difference between control and atherosclerotic arteries was distinctly separated along with the first principal components (PC1) axis, whereas, non-diabetic and diabetic atherosclerotic arteries were obviously discriminated along with the second principle components (PC2) axis. S2 Table shows factor loading values for PC1 and PC2 in score plots. Metabolites with highest absolute factor loading values in PC1 comprised 2-hydroxyglutaric acid, Ala, Ser, Thr, Ile, Asn, Asp and Gly-Asp, which are mainly involved in amino acid metabolism. On the other hand, those in PC2 were phosphoenolpyruvic acid, dihydroxyacetone phosphate, betaine aldehyde+H_2_O, glucose 6-phosphate, fructose 6-phosphate, 2-deoxyglucose 6-phosphate, o-hydroxybenzoic acid, glyceraldehyde 3-phosphate, and UMP, GDP-mannose, CMP and GMP, which are mainly involved in glycolysis/glycogenesis and purine/pyrimidine metabolism.

**Fig 2:**
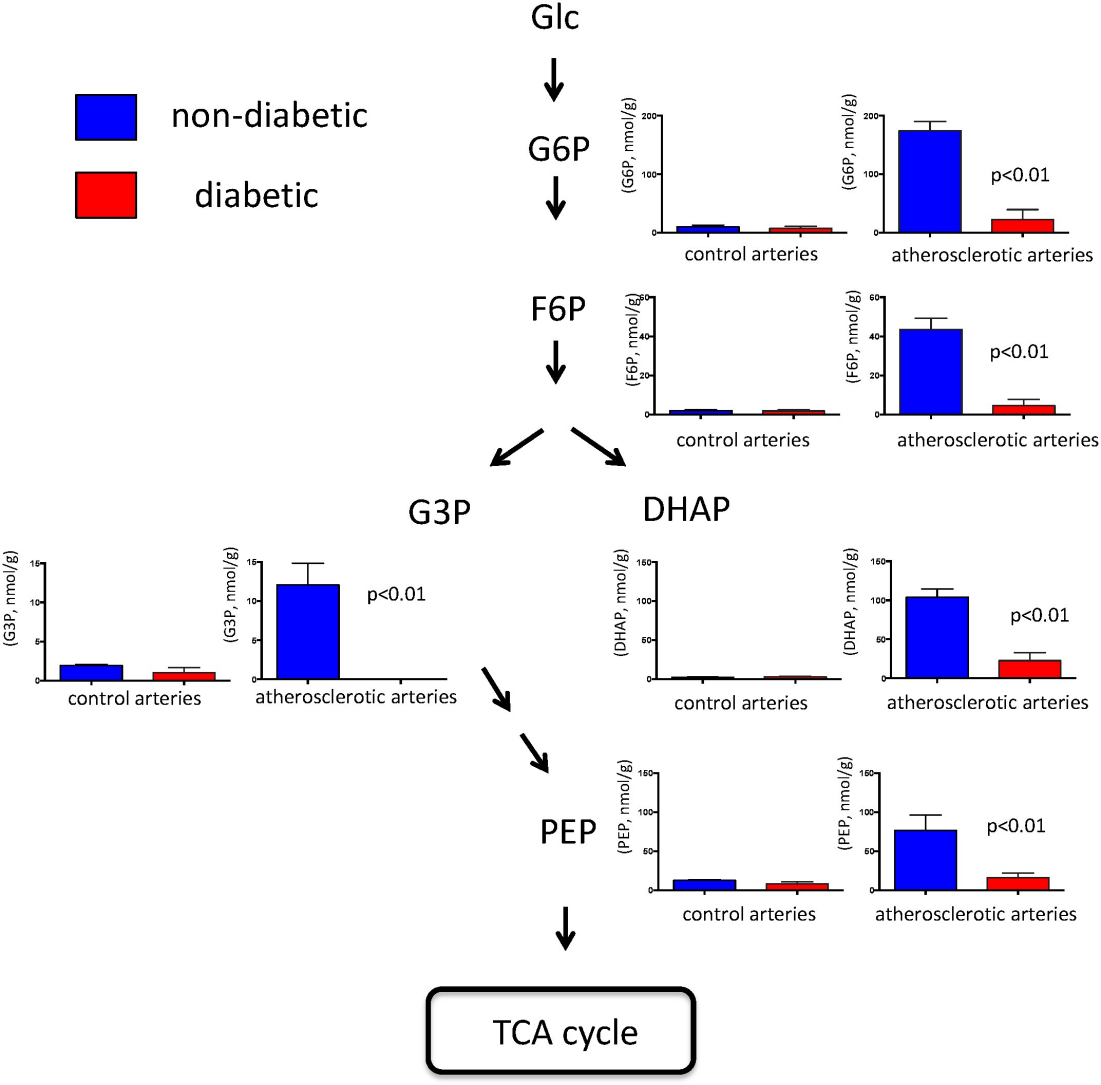
Levels of metabolites associated with glycolysis pathway in arteries of rabbits with and without alloxan-induced diabetes. Metabolite levels are expressed as nmol/g. Control arteries, n = 4; atherosclerotic arteries, n = 5 (Mann Whitney U-test). DHAP, dihydroxyacetone phosphate; F6P, fructose 6-phosphate; Glc, glucose; G3P, glyceraldehyde 3-phosphate; G6P, glucose 6-phosphate; PEP, phosphoenolpyruvic acid.

The results of the hierarchical clustering analysis (HCA) are represented as a heat map (Fig 1B, S3 Table). The results of HCA combined with those of PCA in the three types of arteries confirmed that UMP, GDP-mannose, CMP and GMP belonged to a metabolite cluster with specifically increased levels in diabetic atherosclerotic lesions. In contrast, phosphoenolpyruvic acid, dihydroxyacetone phosphate, betaine aldehyde + H_2_O, glucose 6-phosphate, fructose 6-phosphate, 2-deoxyglucose 6-phosphate, o-hydroxybenzoic acid, glyceraldehyde 3-phosphate belonged to a metabolite cluster with specifically reduced levels in diabetic atherosclerotic lesions (S2, S3 and S4 Tables, Fig 2). Levels of glycolytic metabolites did not significantly differ in control arteries from rabbits with or without diabetes (Fig 2, S4 Table).

### Glucose uptake in diabetic and non-diabetic atherosclerosis in rabbits

The metabolomic analysis confirmed that the glycolytic pathway is altered in diabetic atherosclerosis. Since we previously found that glucose uptake in atherosclerotic lesions is enhanced by hypoxia [15], we examined glucose uptake and hypoxia in diabetic and non-diabetic atherosclerotic lesions using ^18^F- FDG and pimonidazole, respectively.

**Fig 3:**
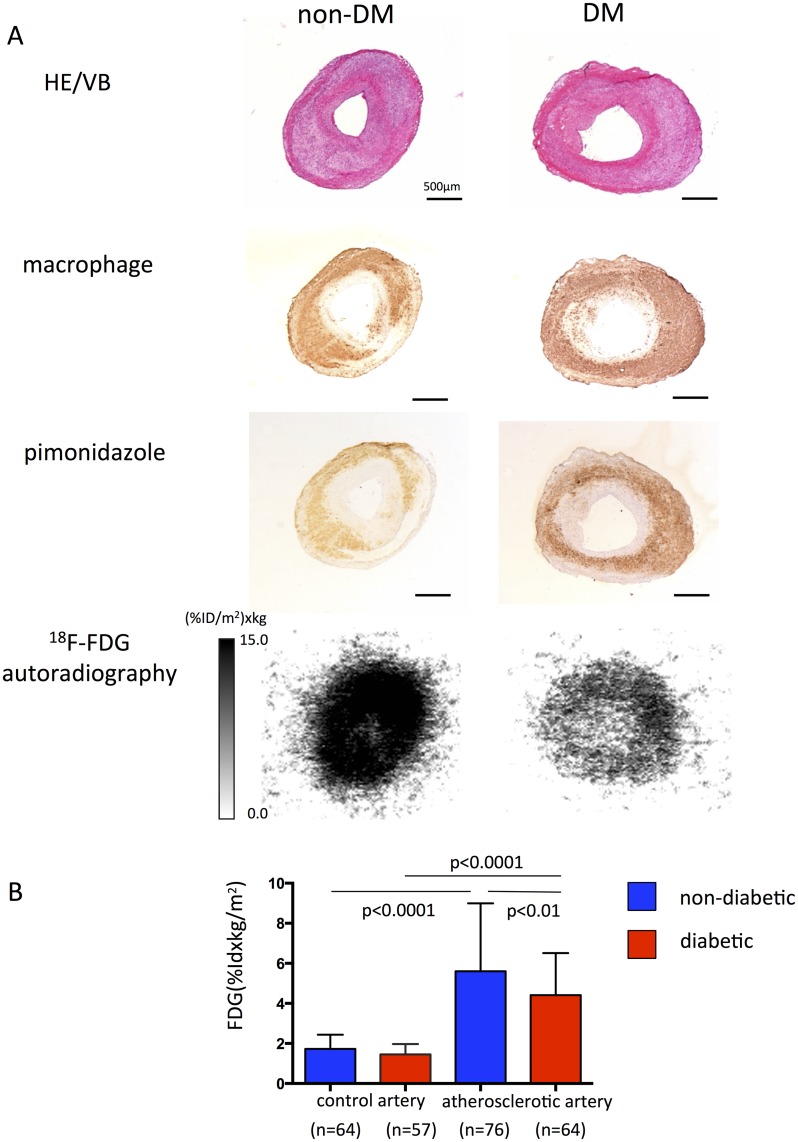
Uptake of ^18^F-FDG in arteries of rabbits with and without alloxan-induced diabetes. A. Representative immunohistochemically stained macrophages, pimonidazole stained with hematoxylin eosin/Victoria blue (HE/VB) and autoradiography of sections of atherosclerotic and control femoral arteries from diabetic and non-diabetic rabbits, respectively. B. Uptake of ^18^F-FDG in control or atherosclerotic arterial sections of diabetic and non-diabetic rabbits (One-way ANOVA with Bonferroni’s multiple comparisons test).

Fig 3A shows autoradiographic and corresponding histological images of atherosclerotic lesions from diabetic and non-diabetic rabbits. Immunohistochemical staining shows hypoxic areas (pimonidazole positive) within macrophage-rich areas located deep in atherosclerotic lesions. The immunopositive areas of hypoxia and macrophage accumulation were larger in diabetic, than non-diabetic atherosclerotic lesions. The SMC area was significantly smaller in diabetic atherosclerotic, than non-diabetic atherosclerotic arteries (Table 1). The ^18^F-FDG uptake in atherosclerotic arteries was significantly higher and larger than those in control arteries from both non-diabetic and diabetic rabbits, but significantly lower in diabetic, than non-diabetic rabbits (Fig 3A and 3B, S4 Table).

**Table 1:** Histological characteristics of control and atherosclerotic arteries in non-diabetic and diabetic rabbits, respectively, fed with 0.5% cholesterol diet.

Histological variables	Non-DM control (n = 64), Atherosclerotic (n = 76) arterial sections	DM control (n = 57), Atherosclerotic (n = 64) arterial sections	P value
Vessel area (mm^2^)			
Control	0.67 ± 0.17	0.63 ± 0.21	0.25
Atherosclerotic	1.50 ± 0.38	1.96 ± 0.50	< 0.0001
SMC area (%)			
Control	90.1 ± 4.6	90.0 ± 5.3	0.95
Atherosclerotic	70.2 ± 15.0	56.7 ± 18.9	< 0.0001
Macrophage area (%)			
Control	0	0	-
Atherosclerotic	23.5 ± 1.8	40.6 ± 1.9	< 0.0001
Pimonidazole area (%)			
Control	0	0	-
Atherosclerotic	11.7 ± 10.3	28.4 ± 15.3	< 0.01

Values are shown as means ± SD and compared between groups using Mann-Whitney u test.

DM, diabetes mellitus; SMC, smooth muscle cells.

### Glucose uptake does not correlate with areas of hypoxia and macrophage infiltration in diabetic atherosclerosis

**Fig 4:**
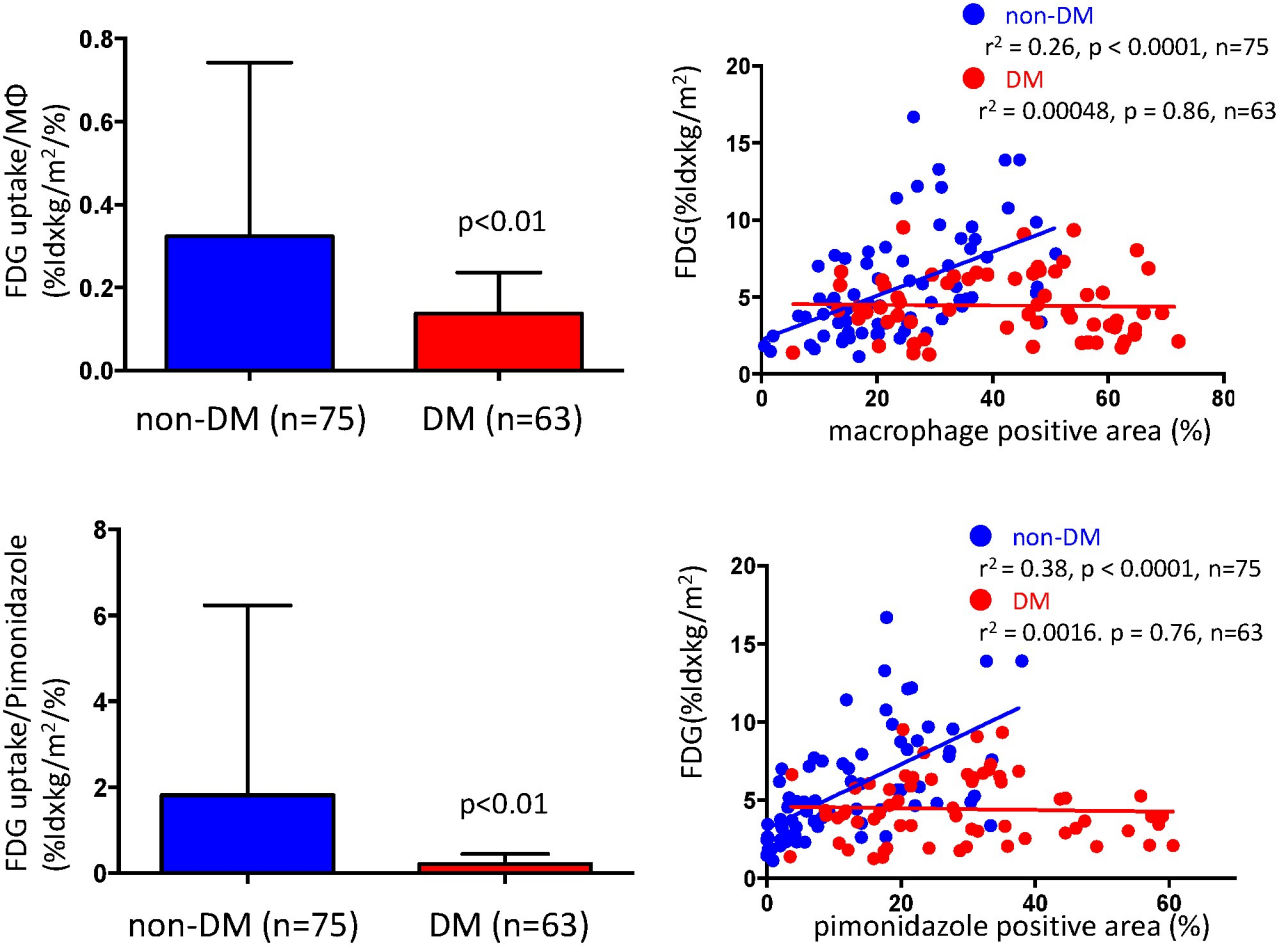
Relationship between ^18^F-FDG uptake and hypoxia in arteries of rabbits with and without alloxan-induced diabetes. Uptake of ^18^F-FDG relative to macrophage or hypoxic areas (Mann-Whitney u test), and correlations between ^18^F-FDG uptake and macrophage infiltration or hypoxic areas (Spearman’s correlation coefficient) in atherosclerotic arterial sections of diabetic and non-diabetic rabbits.

We examined whether or not glucose uptake correlates with macrophage infiltration or hypoxic areas in diabetic atherosclerosis. The uptake of ^18^F-FDG correlated positively with both macrophage accumulation and hypoxic areas in non-diabetic atherosclerotic lesions, which was similar to our previous findings [15]. On the other hand, ^18^F-FDG uptake was significantly lower and did not correlate with either of these factors in diabetic rabbits (Fig 4, S4 Table).

### Reduced nuclear translocation of HIF-1α in diabetic atherosclerosis

**Fig 5:**
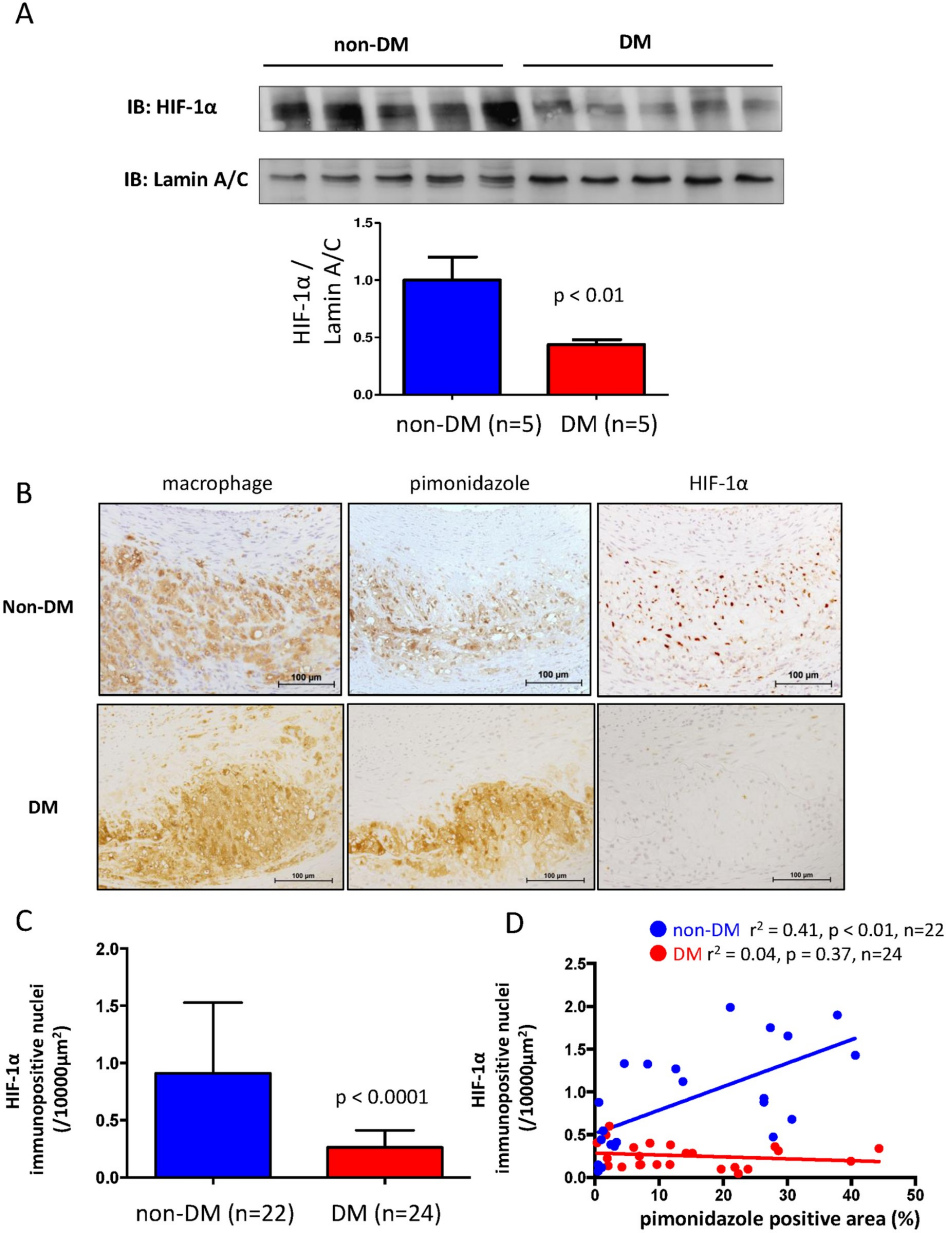
Expression of HIF-1α in atherosclerotic arteries of rabbits with and without alloxan-induced diabetes. A. Western blots of HIF-1α in nuclear extract of atherosclerotic arteries of diabetic and non-diabetic rabbits (Mann-Whitney U-test). Data are shown as fold change relative to non-DM and expressed as mean ± SD. B. Representative immunohistochemical images of macrophages, pimonidazole and HIF-1α in atherosclerotic arteries from diabetic and non-diabetic rabbits. C. Numbers of HIF-1α immunopositive nuclei in atherosclerotic arterial sections of diabetic and non-diabetic rabbits (Mann-Whitney u test). D. Correlations between pimonidazole-immunopositive areas and numbers of HIF-1α immunopositive nuclei in sections of atherosclerotic arteries in diabetic and non-diabetic rabbits (Spearman’s correlation coefficient).

We assessed the nuclear translocation of HIF-1α to evaluate cellular responses to hypoxia in rabbit arteries. Both the HIF-1α content in nuclear extracts and the number of immunopositive nuclei for HIF-1α were significantly reduced in diabetic, compared with non-diabetic atherosclerotic arteries (Fig 5A–5C, S4 Table). The number of HIF-1α positive nuclei correlated positively with hypoxic areas in non-diabetic, but not in diabetic atherosclerotic arteries (Fig 5D). There were no immunopositive nulcei for HIF-1α in non-atherosclerotic control arteries in diabetic or non-diabetic rabbits.

### Enhanced apoptosis in hypoxic areas of diabetic atherosclerotic lesions

**Fig 6:**
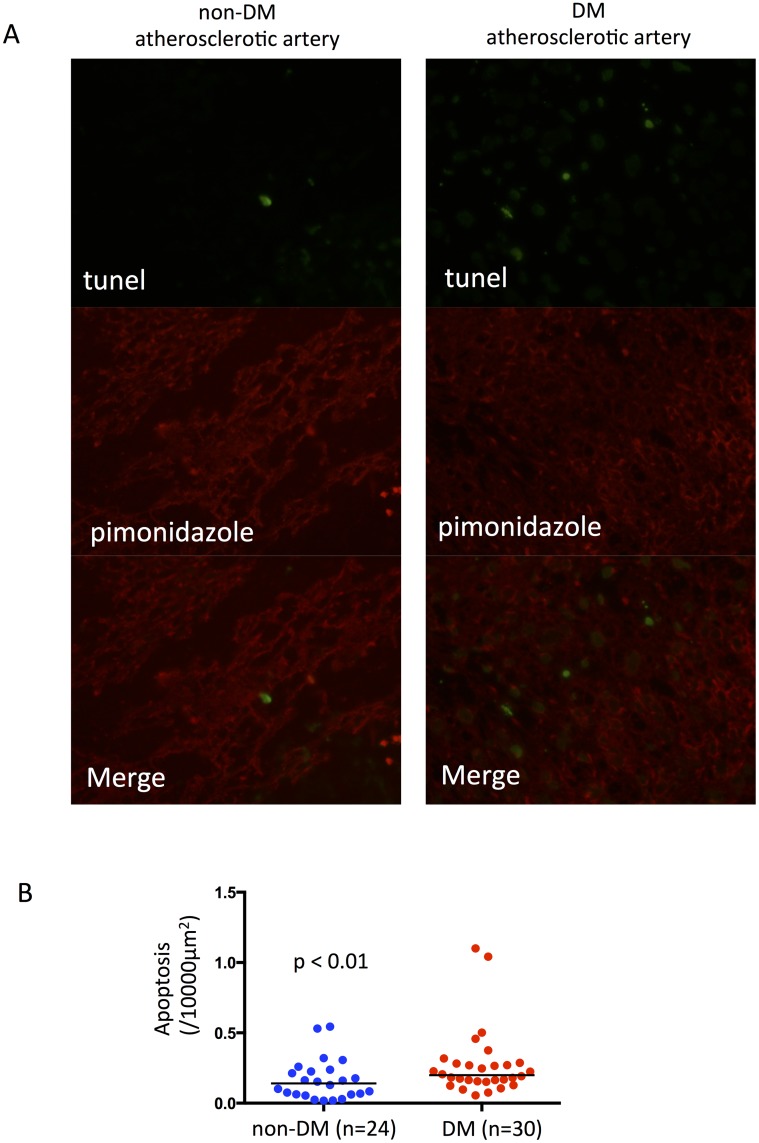
Apoptosis and hypoxic areas in diabetic and non-diabetic atherosclerosis. A. Representative double immunofluorescence staining of hypoxic areas and apoptotic cells in diabetic and non-diabetic atherosclerosis. Images are stained with fluorescein isothiocyanate-labeled anti-BrdU antibody (green), Dylight^™^549 fluorophore-labeled anti-pimonidazole antibody (red), and merged. B. Numbers of apoptotic cells in diabetic and non-diabetic atherosclerosis (Mann-Whitney U-test).

We determined numbers of apoptotic cells in atherosclerotic lesions using TUNEL assays. Double immunofluorescence emission revealed significantly more apoptotic cells in hypoxic (pimonidazole-positive) areas of atherosclerotic lesions from diabetic, than non-diabetic rabbits (Fig 6A and 6B, S4 Table). The number of apoptotic cells significantly correlated with the vascular area (r = 0.64, p<0.001) and macrophage area (r = 0.46, p<0.05) in non-diabetic atherosclerotic arteries, but not in diabetic atherosclerotic arteries (vascular area, r = 0.004, p = 0.98, macrophage area, r = -0.75, p = 0.70). The result suggests the difference in contributing factors for apoptotic cell death in diabetic and non-diabetic atherosclerotic arteries.

## Discussion

We found altered levels of metabolites associated with the glycolysis/glycogenesis and nucleotide intermediate pathways, reduced ^18^F-FDG uptake, impaired HIF-1α regulation of hypoxia and increased numbers of apoptotic cells in atherosclerotic lesions from rabbits with alloxan-induced DM.

Atherosclerosis is the principle cause of mortality among patients with types 1 and type 2 DM. Although epidemiological data have established that DM accelerates atherosclerosis and CVD, the underlying mechanisms remain obscure [16]. Therefore, we developed a rabbit model of diabetic atherosclerosis by combining balloon-induced injury, alloxan injection and a high-cholesterol diet. The lesions were larger and more macrophage-rich in the rabbits with, than without DM. Since alloxan damages pancreatic β cells, the developed DM condition was insulin-dependent type 1 DM [17]. Its condition has been also observed in end-stage type 2 DM [18]. Our model developed hyperglycemia along with obviously increased plasma levels of T-cholesterol and triglyceride. Plasma levels of lipoprotein and plaque formation are increased in several mouse models of type 1 DM [16,19]. Since patients with type 1 DM are frequently associated with dyslipidemia, our animal model simulates atherosclerosis in type 1 DM with dyslipidemia.

The histological features of human diabetic vascular tissue have been previously investigated. A post-mortem study found a larger plaque burden, increased macrophage infiltration and a necrotic core in coronary arteries of patients with types 1 and 2 DM [5], and another study of coronary atherectomy specimens also found more lipid-rich atheroma and macrophage infiltration in patients with DM [20]. The histological features of our animal model were compatible with these findings in patients with DM.

Our metabolomic analysis highlighted 12 metabolites that differed between diabetic and non-diabetic atherosclerosis. These comprised six glycolysis/glycogenesis metabolites, four nucleotides, betaine aldehyde and o-hydroxybenzoic acid. Levels of metabolites associated with glycolysis/glycogenesis were notably decreased in rabbits with diabetic, compared with non-diabetic atherosclerosis. Although few studies have examined metabolic pathways in diabetic vascular tissue, Filla et al. [21] found increases in all metabolites associated with glycolysis and in half of those associated with the tricarboxylic acid cycle, as well as methylglyoxal and amino acids in the non-atherosclerotic aorta in diabetic mouse (db/db). The results are not comparable of those of the present study. The inflammatory and hypoxic microenvironments in the atherosclerotic lesions affect metabolic status of these cells [15]. The metabolic differences might have been due to differences in diabetic models and the presence of atherosclerotic lesions in our model. Peiró et al. [22] reported that high glucose enhances activities of glucose-6-phosphate dehydrogenase, a pentose phosphate pathway enzyme, and nicotinamide adenine dinucleotide phosphate oxidase by interleukin 1β in cultured SMCs. Although metabolite levels of pentose phosphate pathway increased in the diabetic and non-diabetic atherosclerotic arteries compared with non-atherosclerotic arteries, the diabetic condition did not potentiate the metabolic change in our in vivo study (S3 Table).

We assessed metabolic changes in glycolysis by measuring ^18^F-FDG uptake and determining its relationship to vascular hypoxia. The uptake of ^18^F-FDG was reduced in diabetic, compared with non-diabetic atherosclerotic lesions. These findings are compatible with the present results of the metabolome analysis. We previously found that increased ^18^F-FDG uptake in macrophage-rich atherosclerotic lesions was positively associated with thrombogenicity [23]. Hyperglycemia itself impairs ^18^F-FDG uptake, reportedly via competition with endogenous blood glucose [24,25], whereas contradictory findings have also been published [26,27]. We found here that ^18^F-FDG uptake in non-injured arteries did not significantly differ between diabetic and non-diabetic rabbits, indicating that reduced ^18^F-FDG uptake in diabetic atherosclerotic lesions is due to metabolic changes in vascular cells rather than competition with endogenous blood glucose. Hellberg et al. [28] found lower absolute ^18^F-FDG uptake and a lower aorta-to-myocardial ^18^F-FDG uptake ratio determined by autoradiography in diabetic, than in non-diabetic mice. The uptake of ^18^F-FDG positively correlated with macrophage content and hypoxic (pimonidazole-positive) areas of atherosclerotic lesions in rabbits without DM, but a correlation was not found in diabetic atherosclerosis. The lack of relationship between ^18^F-FDG uptake and macrophage content in diabetic atherosclerosis suggests alteration of macrophage glucose metabolism in alloxan-induced diabetic rabbits.

The promotion of glucose uptake and the glycolytic pathway by hypoxia is generally HIF-1-dependent in many types of cells including macrophages [15]. We found reduced HIF-1 expression in diabetic atherosclerotic lesions, and this did not increase as the size of hypoxic areas increased. Since HIF-1 signaling protects against cell death and apoptosis by reducing oxidative stress, mitochondrial injury and metabolic derangement [29], the inappropriate response of HIF-1 to hypoxia associated with diabetes would induce cell death and increase the necrotic core. A study of wound healing in diabetic mice model found reduced levels of HIF-1α and HIF-1 target genes in lesions despite hypoxia [30]. Taken together, these and the present findings suggest that hypoxic responses are impaired in diabetic atherosclerosis, and that reduced HIF-1α nuclear translocation partly contributes to altered glucose metabolism, macrophage accumulation and enhanced apoptosis in atherosclerotic lesions.

Betaine aldehyde, intermediate metabolite of choline and betaine, belonged to the metabolite cluster with reduced levels in diabetic atherosclerosis. Betaine is an essential osmolyte and methyl group donor [31]. Its metabolism links several metabolites, including choline, homocystine, and methionine. Clinical studies showed that plasma betaine levels were negatively associated with triglyceride and non-high-density lipoprotein cholesterol, and that lowest quintile of plasma betaine was associated with secondary acute myocardial infarction [32,33]. Although exact mechanisms are unclear, the evidence suggests that alteration of betaine metabolism is associated with diabetic atherosclerotis and atherothrombotic complication.

This study has several limitations. The metabolites investigated herein represent only a portion of the total vascular metabolites. Further analysis by CE-TOFMS combined with liquid chromatography-mass spectrometry might unveil further comprehensive metabolic changes. We analyzed specimens at only five weeks after inducing diabetes and thus time-course changes in vascular metabolism during atherogenesis were not investigated. Alloxan-induced diabetes is a model of type 1 DM, but type 2 DM is a more common risk factor for CVD. Studies of type 2 DM models should provide further understanding of the underlying mechanisms of diabetic atherosclerosis.

In conclusion, our results suggest that glycolytic metabolism and glucose uptake are altered in diabetic atherosclerosis. An altered hypoxic response might partly contribute to metabolic changes and enhanced apoptosis in diabetic atherosclerosis.

## Supporting information

S1 Table Body weight and serum parameters in non-diabetic and diabetic rabbits fed with 0.5% cholesterol diet at baseline and five weeks later. (DOCX)

S2 Table Factor loading value for principal component 1 and 2 in arterial metabolites. (XLSX)

S3 Table Hierarchical clustering analysis of arterial metabolites. (XLSX)

S4 Table Original data of Figs 2, 3B, 4, 5 and 6B. (XLSX)
