# Supplementary material for: Altered glucose metabolism and hypoxic response in alloxan-induced diabetic atherosclerosis in rabbits
Source: PLoS One. 2017 Apr 14;12(4):e0175976. doi: 10.1371/journal.pone.0175976 (PMC5391952; doi:10.1371/journal.pone.0175976)
Supplement: S1 Table — (DOCX) [file pone.0175976.s001.docx]

**Additional table 1.**

**Body weight and serum parameters in non-diabetic and diabetic rabbits fed with 0.5% cholesterol diet at baseline and five weeks later.**

|  | Non-DM (n = 10)  (means ± SD) | DM (n = 10)  (means ± SD) | p value |
| --- | --- | --- | --- |
| Body weight (kg) |  |  |  |
| 0 week | 3.05 ± 0.25 | 2.94 ± 0.05 | 0.28 |
| 5 weeks | 3.31 ± 0.24 | 3.05 ± 0.22 | 0.02 |
|  |  |  |  |
| Glucose (mg/dL) |  |  |  |
| 0 week | 110 ± 10 | 111 ± 12 | 0.85 |
| 5 weeks | 106 ± 18 | 368 ± 55 | <0.0001 |
|  |  |  |  |
| Insulin (μIU/mL) |  |  |  |
| 0 week | 6.08 ± 3.92 | 4.36 ± 3.97 | 0.34 |
| 5 weeks | 4.48 ± 2.40 | 1.34 ± 1.17 | 0.002 |
|  |  |  |  |
| Total cholesterol (mg/dL) |  |  |  |
| 0 week | 23.1 ± 10.0 | 25.6 ± 10.4 | 0.59 |
| 5 weeks | 811.8 ± 374.3 | 1644.0 ± 626.9 | 0.002 |
|  |  |  |  |
| Triglyceride (mg/dL) |  |  |  |
| 0 week | 24.5 ± 19.7 | 19.7 ± 10.2 | 0.5 |
| 5 weeks | 15.8 ± 11.9 | 130.0 ± 11.9 | 0.026 |

DM, diabetes mellitus.

Data were analyzed with Mann-Whitney u-test.
